# The politics of breastfeeding: a feminist analysis of breastfeeding policies and promotion in Nigeria

**DOI:** 10.1186/s13006-026-00824-x

**Published:** 2026-03-11

**Authors:** Vera Oko

**Affiliations:** Faculty of Social Sciences, Institute of Feminist and Gender Studies, University of Ottawa, Ottawa, ON Canada

**Keywords:** Breastfeeding, Infant feeding policies, Nigeria, Maternal

## Abstract

**Background:**

Since the 1990s, global health policies have prescribed breastfeeding as an ideal and primarily positive practice essential to child survival and maternal health. In Nigeria, infant feeding policies have largely drawn on these global frameworks in promoting exclusive breastfeeding as a strategy against infant mortality.

**A feminist analysis of breastfeeding policies and promotion in Nigeria:**

Grounded in feminist ethics of care and the Ubuntu philosophy, this critique examines the contradictions between breastfeeding policies and maternal realities in Nigeria. The analysis identified the following: (1) a universalist approach in global and Nigerian breastfeeding policies that assumes breastfeeding is ideal and positively experienced by most mothers. (2) policies place breastfeeding at the top of the invisible hierarchy of infant feeding and pathologize quotidian maternal experiences by framing exclusive breastfeeding as ideal, and positive in most cases thereby marginalizing the challenges and costs many mothers face. (3) unaddressed gaps between policy directives and structural conditions in Nigeria such as underdeveloped social amenities, insufficient maternity leave laws and cultural realities, reinforce gender inequalities, erode maternal autonomy and influence breastfeeding practices.

**Conclusion:**

The gaps between breastfeeding policies and maternal realities in Nigeria highlight the need for policies discourse that are grounded in considering the challenges mothers face in breastfeeding. The review suggests ways to move beyond frameworks that treat infant care as solely a mother’s responsibility by emphasizing the essential role of support in infant care.

**Clinical trial number:**

Not applicable N/A.

## Background

Since the 1990s, the World Health Organization (WHO) and the United Nations Children’s Fund (UNICEF) have promoted breastfeeding as the optimal form of infant feeding, framing it as an ideal and mainly positive practice that benefits both infants and mothers [1]. In response, the Nigerian Ministry of Health implemented policies such as the National Policy on Infant and Young Child Feeding to promote exclusive breastfeeding (EBF) and reduce infant mortality, primarily through educating mothers on how to breastfeed [2].

Despite widespread knowledge and willingness to breastfeed, many Nigerian mothers introduce complementary foods or stop breastfeeding before six months, contributing to a national exclusive breastfeeding rate of 34%, below the World Health Assembly target of 50% [1, 2]. This gap between policy expectations, maternal intentions, and actual practices highlights that there may be other intersectional factors that influence breastfeeding.

Hence this analysis of breastfeeding policies in Nigeria, examines how policies marginalize maternal realities [3]. It further shows how breastfeeding promotion largely obscures factors that influence the practice such as structural realities, insufficient maternal leave and limited access to clean water and electricity. Furthermore, the analysis also considers how policies reinforce cultural norms that can erode maternal autonomy. Drawing on the Ubuntu philosophy [4] and feminist ethics of care [5] This review emphasizes the need for collective support for nursing mothers from both partners and the state. By situating breastfeeding within these broader structural and feminist perspectives, the critique contributes to debates on infant feeding policies and breastfeeding.

## Analyzing breastfeeding through feminist theoretical contexts

Feminist theory, broadly speaking, seeks to highlight the experiences of women and other marginalized groups within broader struggles for social justice [6]. This review draws on Chisale’s feminist theorising of the philosophy of Ubuntu, which means “I am because you are” and its emphasis on human interconnectedness [4]. Ubuntu is rooted in African communal values and highlights our relationships and interconnection with others, God, ancestors, self. Applying this philosophy to caregiving, Chisale proposes an ungendered Ubuntu and encourages shared responsibility among men, women, and society, challenging the idea that care work is only women’s responsibility. Ungendered ubuntu is a feminist reinterpretation of ubuntu philosophy that emphasizes shared caregiving and community support without assigning care work based on gender [4]. This perspective is particularly relevant for breastfeeding promotion in countries like Nigeria, where unpaid caregiving labor is largely considered as women’s work including breastfeeding. However, this analysis acknowledges that not all individuals who breastfeed identify as women.

In addition, breastfeeding and policy efforts in Nigeria is analysed in this review, through the feminist ethics of care which expands the idea of caring beyond the private sphere. The Ethics of care is a feminist moral framework highlighting relationships, interdependence, and the moral significance of care as both a private and social responsibility, where all genders and the state can provide support [5]. Philosopher Virginia Held argues that care should be a social value, supported not just by individuals but also by governments and society [5].

This approach challenges neoliberal thinking, which emphasizes individual responsibility over collective support [7]. Neoliberalism in the context of breastfeeding promotion and policies, place pressure on mothers to make the “right” choices which is considered to be breastfeeding, regardless of structural realities and the career, physical, mental, emotional costs, borne by mothers in their breastfeeding journey [8].

### Feminist perspectives on breastfeeding

Feminist debates on breastfeeding have long highlighted tensions between maternal autonomy and advocacy for infant feeding. Some feminists such as Penny Van Esterik, emphasize the importance of protecting women’s reproductive agency and rights [9]. However, other feminists argue that policies framing infant survival as dependent solely on breastfeeding place the burden of reproductive labor on mothers, who often also engage in productive labor, thereby exploiting women’s bodies and limiting their autonomy [10]. Productive labor is sometimes substituted with ‘paid work’ and refers to work that is recognized in the formal economy and compensated with payment [11]. While Reproductive labor refers to unpaid labor that sustains households and caregiving such as breastfeeding [12, 13]. Some feminist scholars emphasise that breastfeeding, is essential to reproducing the workforce, yet remain an undervalued form of labor expected of and borne by women which contribute to persistent gender inequalities [10, 14].

Gender inequalities are systematic disparities in power, resources, opportunities, and treatment based on gender [3]. In breastfeeding, these inequalities manifest when women disproportionately bear unpaid care work and face structural barriers to combining paid work with intensive caregiving [3, 10, 11]. These inequalities might unfold in the way that a woman loses hours of pay or half of her pay due to reproductive labor [10]. Additionally, feminists agree that intimate partner and spouse involvement in household and childcare tasks, while not directly assisting with breastfeeding, may significantly support maternal well-being [3]. Despite these insights, there remains a paucity of feminist analysis of breastfeeding in Nigeria [11]. This review addresses the gap by examining how Nigerian policies intersect with women’s realities. The examination of policy and challenges in breastfeeding, highlights constraints and potential strategies to support mothers in their infant feeding journeys.

## Global infant feeding policies and breastfeeding challenges

Aggressive marketing tactics by infant formula companies, alongside the Nestlé boycott amid unprecedented infant mortality rates, particularly in developing countries, ushered in international policy instruments for promoting breastfeeding [15]. First, *the International Code of Marketing of Breastmilk Substitutes* was adopted at the 34th World Health Assembly in 1981 [16]. One primary purpose of the code was to prohibit unethical formula advertising which increased the positioning of breastfeeding as the most ideal form of infant feeding. Subsequent policies sought to increase breastfeeding practices and reduce complementary feeding practices, including *the 1990 Innocenti Declaration*, which further recognized breastfeeding as a fundamental right of infants [17]. Additionally, these policies emphasised that breastfeeding remains mainly positive and beneficial to both mother and baby. Furthermore, despite feminist advocacy against the medicalizing women’s health and bodies, the *Baby-Friendly Hospital Initiative (BFHI)* was launched by WHO and UNICEF in 1991 [3]. This policy was designed to encourage new mothers to initiate and continue breastfeeding through hospital-based programs. Furthermore, global health infant feeding policies recommends exclusive breastfeeding for at least 6 months, to be continued with complementary feeding for two years [1, 2]. Exclusive Breastfeeding is defined as feeding an infant only breast milk, with no other liquids or solids except vitamins, minerals, medicines, or ORS [1–3]. Together, these policies reinforce the attainment of exclusive breastfeeding as a public health priority.

Furthermore, the breastfeeding policies which promote exclusive breastfeeding as the optimal infant feeding practice, do so within what I call the invisible hierarchy of infant feeding. This invisible hierarchy is implied in the unspoken ranking of infant feeding practices. The ranking positions exclusive breastfeeding at the top of the pyramid while other infant feeding practices, such as complementary feeding is ranked lower. However, there are several problematic elements in this positioning of exclusive breastfeeding suggested within policy discourse of breastfeeding.

First, the framing of breastfeeding as the ideal practice which all mothers are to perform without question to secure a baby’s health, draws on a culture of risk where women are pressured to minimize every possible risk to their infants by breastfeeding even at the expense of personal, financial, emotional or physical costs [3, 8, 10, 18]. Further, these policies act as a disciplinary tool of policing the bodies of mothers through rewarding compliance with the tag of ‘good mother’ and punishing the absence of breastfeeding with labels of ‘failure’ at breastfeeding or a ‘bad mother’ [18, 19]. Hence, turning to formula or other infant feeding methods that may be necessary for child survival due to specific maternal health needs and circumstances becomes a less desirable even when breastfeeding is not possible due to maternal health complications [3, 8].

Secondly, policy narratives of exclusive breastfeeding that frame the practice as positive and best, as evident in page 7 and 13 of the Innocenti declaration and reflected in page 9 of the Nigerian infant feeding policy [17, 20], often overlook the reality that many mothers do not experience breastfeeding as ideal or positive [3]. Furthermore, the policies risk portraying normal breastfeeding experiences as rare or abnormal, a phenomenon I term the pathologizing of the quotidian. This occurs when challenges in breastfeeding such as insufficient lactation, mastitis, clogged ducts, painful or cracked nipples, are treated as deviations or failures rather than normal maternal realities [3, 8]. For instance, a study in Jos, Nigeria, found that 51% of mothers were medically unable to exclusively breastfeed due to illness or health conditions [21]. Here, more than half of the mothers turned to formula or supplemental feeding which becomes not only ideal in such cases but also preserves the lives of the babies whose mothers cannot breastfeed. Thus, as Professor of sociology, Phyllis Rippey notes, while breastfeeding is crucial for infant health, without formula or supplemental feeding some babies will die [3].

Furthermore, Ogbonna et al.’s 2018 qualitative study found that while some mothers experience joy in breastfeeding, many face difficulties and pains including breast engorgement, pain, sadness and frustration. One mother said, “You just find yourself crying sometimes, one child is just demanding so much from you” and another recounted, “My breasts were full, the pain was so much. It was horrible” [8]. In the study, some mothers expressed turning to God, spirituality and accepting help with domestic chores from their mother in-laws. Despite, these struggles in breastfeeding reported by many mothers which further reflects the importance of Ubuntu’s emphasis on interdependence especially during challenges, breastfeeding is still largely described as solely positive in policies thereby systematically erasing the challenges of the practice commonly experienced by many mothers globally and in Nigeria. Consequently, breastfeeding policies reflect universalist frameworks through generalising the positive ideals of breastfeeding without adequately highlighting regularly experienced breastfeeding challenges. Furthermore, global health breastfeeding policies have been adopted by many countries globally, including Nigeria.

## Breastfeeding policy and practices in Nigeria

Nigeria is a west African country with an estimated 227 million people in 2023 [22]. The country faces high infant mortality rates shaped by structural, cultural, and socio-economic factors [2]. Furthermore, since the 1990s, diarrhea has been named as a leading cause of infant deaths, with breastfeeding promoted as the solution [2]. Hence, Nigeria adopted World Health Organization WHO and the United Nations Children’s Fund, UNICEF policies to combat infant mortality which includes the National Policy on Infant and Young Child Feeding [20, 24]. This policy promotes exclusive breastfeeding to improve child survival rates It affirms all infants’ right to six months of exclusive breastfeeding and complementary feeding up to two years, by emphasizing the education and encouragement of mothers to breastfeed [24]. Unlike Indonesia, where legal measures protect breastfeeding [3], Nigeria imposes no penalties for not practicing breastfeeding. However, Nigeria have aligned with global policy guidelines to position exclusive breastfeeding as optimal amongst infant feeding choices [2, 24]. The national policy on Infant and Young Child Feeding, mandates exclusive breastfeeding unless medically contraindicated, reinforcing societal expectations that mothers breastfeed under nearly all circumstances [20]. The policy is geared towards increasing breastfeeding through efforts such as training of health care workers to inform mothers about practicing breastfeeding.

Despite the adaptation and efforts to implement the National Policy on Infant and Young Child Feeding policy, Nigeria’s exclusive breastfeeding rates remain modest compared to other West African countries and global landscapes [2]. Furthermore, some comparative studies on breastfeeding trends in Nigeria over the years suggest a slight increase in breastfeeding since policy implementation. For instance, Ogbo et al.’s 2017 paper analyzed trends from 1999 to 2013 and found modest gains following relevant policy implementation in Nigeria. Additionally, the exclusive breastfeeding rates have rising from 23% in 2019 to 34% in 2024 [1, 25]. Such increase over the years raises some suggestions that policy efforts have increased breastfeeding rates not only in Nigeria but in other countries where some increment in breastfeeding have been recorded since global health policies emerged [1]. However, since breastfeeding is complex and shaped by intersectional factors such as culture, structural realities and maternal socio-economic conditions [3, 18], it is unlikely that policy alone is responsible for the increase in breastfeeding rates.

Furthermore, it has been argued that medical training, hospital-based education, and media campaigns contribute to increases in exclusive breastfeeding rates, particularly within the first hour after birth [2, 25, 26]. Policies such as the Baby-Friendly Hospital Initiative (BFHI), adopted in 1991, promote early initiation of breastfeeding [25, 27]. However, research on breastfeeding in Nigeria show that most Nigerian mothers already have substantial knowledge of breastfeeding and its benefits, yet knowledge rarely translates to sustained breastfeeding [2, 26]. Literature on the practice of breastfeeding in Nigeria have linked the interruption of the practice to mothers’ return to work and insufficient support from spouse [2, 26].

From a feminist perspective, breastfeeding promotion policies, adverts and medical training that promotes early initiation of breastfeeding in hospitals may inadvertently reinforce normative assumptions that all mothers both desire and are physically able to breastfeed obscuring challenges they face while limiting space for maternal autonomy [10, 14, 28].

Interestingly, home births particularly in Northern Nigeria often involve practices, such as discarding colostrum or giving infants water or herbs, which can cause infections and increase infant mortality [2]. However, research shows that mothers who deliver in hospitals are more likely to initiate breastfeeding early and avoid these practices [2, 25]. In this context, policy and health promotion efforts have helped to change some practices that conflict with performing breastfeeding in the absence of maternal challenges.

## Gaps between policy directives and breastfeeding realities in Nigeria

Although breastfeeding is often framed as a cost-free infant feeding method, the gap between policy promotion, structural support and breastfeeding rates in Nigeria exposes a fundamental flaw in this line of logic. In Nigeria, maternity leave provisions are insufficient. The Labour Law Act grants only 12 weeks at 50% pay and excludes mothers employed less than six months [24]. This makes it difficult to meet the six-month exclusive breastfeeding recommendation, forcing many mothers to discontinue breastfeeding upon returning to work [24, 26]. Furthermore, the maternity law protection mainly benefits formally employed women, a small fraction of Nigeria’s female workforce, as 95% of women work in the informal sector [23, 24]. Without extending breastfeeding-friendly policies beyond the formal labor market, most working mothers lack support, leaving them to navigate competing demands between policy expectations and economic survival, where maintaining income often becomes the personal financial cost of breastfeeding borne by mothers [10].

The absence of basic social amenities, such as access to clean water and reliable water supply, adds a significant burden for many Nigerian mothers with infants. Clean and convenient water impacts safety of supplementary foods and maternal labor. However, despite abundant water resources, approximately 58 million people lack access to safe water in Nigeria with 71% of rural and 42% of urban residents affected [29–31]. Furthermore, women bear a disproportionate share of household water provision. For example, in Abuja, 51% of households identified women and female children as primary water fetchers, spending 1–2 h daily, up to 4 h in the dry season, collecting water [29, 30] This reduces time for income-generating work, domestic duties, rest, and breastfeeding [40. Limited state and partner support for these labor-intensive tasks reinforces entrenched gender inequalities [29, 30]. Furthermore, Access to clean water is crucial not only because it fuels gender inequalities and impacts maternal health [30] but also because it influences the ability to make safe formula and infant foods, thereby posing the potential to impact infant health as well [10]. Additionally, even in the more developed areas in Nigeria, with some access to clean water or tap water, the problem of electricity and constant energy and refrigeration remains a challenge that can affect infant feeding and infant health. Household access to electricity in Nigeria remains critically low, with approximately 60% of the population, over 80 million people, lacking electrical service [31]. Consequently, the positioning of breastfeeding as the solution to infant feeding in policies risks leaving deeper issues of water access and electricity unaddressed by the state governments. Without social amenities, infrastructure and support, breastfeeding policy and infant health goals remain unsustainable in Nigeria.

## Intersections between policy and culture

The Nigerian culture embraces breastfeeding [8]. Furthermore, fathers who are the patriarchal head of the house make decisions on breastfeeding based on cultural expectations and trust in medical recommendations to breastfeed from the hospitals [2]. Hence, partners often make key decisions in the family and enforce exclusive breastfeeding. Sometimes, the decisions partners make towards breastfeeding are irrespective of mothers’ preferences, for example, one participant in Joseph and Earland’s (2019) study of breastfeeding mothers in northern Nigeria, notes that: “. When I told my husband what the health workers say about exclusive breastfeeding, he [the husband] said it was ok; he permitted me to go ahead.” Furthermore, in a study on breastfeeding mothers’ experiences in the Southwest of Nigeria by Ogbonna et al. (2018), one respondent explains how her husband restricted her from stopping the practice of breastfeeding. She notes: *“*I wished to discontinue breastfeeding after eight months. However, my husband refused. He said I should breastfeed for one year. I felt I needed my freedom, so I wanted to stop, but he said I had to continue” [8]. Hence, when policy and the Nigerian culture meet, they reinforce one another within existing gendered power relations, that erodes maternal autonomy in breastfeeding. In this dynamic, the ‘choice’ to breastfeed, is therefore an illusion for most Nigerian mothers who encounter limited spaces to exercise their agency between the hospitals and the home front. As a participant in the Ogbonna et al.’s 2018 study notes: “…For me, it was never even an option ‘not’ to breastfeed. Especially for an African woman, from the outset, it is just as if it is part of our psyche to breastfeed” [8].

Interestingly, Nigerian mothers have developed indigenous strategies to cope with the demands of productive and reproductive work. One such tradition is called the Omugwo [32]. The Omugwo is an Igbo practice of postpartum care, where Grandmothers or extended family live with new mothers, often for up to three months, to assist with infant care, household chores, and cooking [8, 32]. Furthermore, the Omugwo provides emotional support, cultural knowledge transfer and nutritional assistance, to new others further allowing mothers to focus on breastfeeding.

### Suggestions for the way forward

Chisale’s feminist re-reading of Ubuntu advocates for the ungendering of Ubuntu which is the “ungendering” of care labor [4]. When this lens is applied to infant care in Nigeria, it translates and equates to the challenging of the patriarchal notion that caregiving, such as diapering, cooking, cleaning, and putting babies to sleep, is primarily a woman’s responsibility. In the context of breastfeeding, ungendering care redistributes caregiving responsibilities, across society, encouraging men and the governments to participate in alleviating the triple weight of productive, reproductive, and care work which most mothers’ bear. It is important to note that while spouses may not assist directly with breastfeeding, and their involvement does not reduce the time mothers spend nursing, its absence has been shown to reduce breastfeeding duration [26] and its presence has been shown to increase breastfeeding duration [33].

Hence, the ungendering of care holds significant implications for maternal support and breastfeeding. For governments, it entails developing social amenities and implementing longer maternal leave policies and welfare that protect mothers in both formal and informal work. rather than relying solely on breastfeeding promotion and mothers to safeguard infant health. In policy terms, this means adopting context-sensitive language and approaches that recognize and embrace difficulties and challenges as normal in breastfeeding. And for spouses, it involves physical support with household chores and childcare duties [33]. Hence, to maximise support for breastfeeding mothers, policies should consider the involvement of partners and the government in providing practical, emotional, and structural assistance.

## Limitations

This study is limited to Nigeria and is based mainly on existing literature concerning breastfeeding, global health, and Nigerian infant feeding policies. As a result, the findings may not be generalizable to other national contexts.

## Conclusion

Decades ago, global health infant feeding policies emerged with the aim of promoting breastfeeding and reducing infant mortality [1, 2]. The Federal Government of Nigeria adopted several policies to promote breastfeeding, including the Infant and Young Child Feeding (IYCF) policy. However, drawing on the ethics of care and the African Ubuntu philosophy [12, 13] this analysis demonstrates that breastfeeding promotion policies often universalise the practice as ideal while largely overlooking the challenges mothers face [2, 10, 24]. This review emphasizes the need for the active involvement of spouses and the government in providing practical support to mothers. By highlighting how factors such as culture and maternal support influence breastfeeding practices, this analysis contributes to broader social debates on breastfeeding.

## Data Availability

No datasets were generated or analysed during the current study.

## Declarations

### Author information

Master’s degree in Women’s Studies. Doctoral Degree in Feminist and Gender Studies (in View).
